# Imaging findings and complications after curettage of atypical cartilaginous tumors in long bones: a retrospective single-center cohort study

**DOI:** 10.1007/s00256-025-05061-7

**Published:** 2025-10-29

**Authors:** Salvatore Gitto, Alberto Soro, Mattia Sica, Valentina Molinari, Domenico Albano, Stefano Fusco, Francesca Serpi, Simone Mazzoli, Gennaro Maria Scotto, Renato Cuocolo, Carmelo Messina, Alessandro Luzzati, Luca Maria Sconfienza

**Affiliations:** 1 Dipartimento Di Scienze Biomediche Per La Salute, Università Degli Studi Di Milano, Milan, Italy; 2 IRCCS Istituto Ortopedico Galeazzi, Milan, Italy; 3 UOC Radiodiagnostica, Presidio San Carlo Borromeo, ASST Santi Paolo e Carlo, Milan, Italy; 4 Department of Medicine, Surgery, and Dentistry, University of Salerno, Baronissi, Italy; 5 Scuola Di Specializzazione in Radiodiagnostica, Università Degli Studi Di Milano, Milan, Italy; 6 Dipartimento Di Scienze Biomediche, Chirurgiche Ed Odontoiatriche, Università Degli Studi Di Milano, Milan, Italy; 7 Department of Radiology, ASST Grande Ospedale Metropolitano Niguarda, Milan, Italy; 8 UOC Radiodiagnostica, ASST Centro Specialistico Ortopedico Traumatologico Gaetano Pini-CTO, Milan, Italy

**Keywords:** Atypical cartilaginous tumor, Chondrosarcoma, Curettage, Enchondroma

## Abstract

**Objective:**

To assess imaging findings and complications after curettage of atypical cartilaginous tumors (ACTs) in long bones.

**Materials and methods:**

This retrospective study included patients with central ACTs of long bones treated with curettage, adjuvants, and cementation or bone grafting, who had clinical and imaging follow-up data available for at least 2 years after surgery. All imaging studies (radiographs, CT, MRI) were independently assessed by three radiologists. Clinical information was collected from the medical records.

**Results:**

Sixty-eight patients were included (median age [interquartile range, IQR], 53 [45–60] years). Bone graft and cement were used in 53 (77.9%) and 15 (22.1%) patients, respectively. Prophylactic internal fixation was performed in 63 (92.7%) patients. The median (IQR) follow-up duration was 42 (30–64) months. Normal imaging findings were identified at follow-up in 45 patients (66.2%). Our reported complications included peri- (2.9%) and post-operative (8.8%) bone fractures, incomplete bone graft integration (24.5%, out of patients treated with bone grafting), cement loosening (26.7%, out of patients treated with cementation), fixation hardware rupture (1.6%) or loosening (6.3%, out of patients treated with internal fixation) and residual disease (1.5%). Incomplete bone graft integration and cement loosening were associated with tumor location in the humerus (p = 0.023). Inter-reader agreement ranged between moderate and excellent (Fleiss’s K = 0.522–1).

**Conclusion:**

After curettage of ACTs in long bones, complications are detected on follow-up imaging examinations in one third of patients, mainly including fractures, incomplete bone graft integration and cement loosening.

**Supplementary Information:**

The online version contains supplementary material available at 10.1007/s00256-025-05061-7.

## Introduction

In the 2020 edition of the World Health Organization (WHO) classification of bone tumors, the term “atypical cartilaginous tumor” (ACT) denotes low-grade cartilaginous lesions of long bones [1]. Cartilaginous lesions with the same histology as ACT, but located in the axial skeleton, are classified as chondrosarcoma grade I [1]. In long bones, the relatively new definition of ACTs reflects their indolent clinical behavior with unlikelihood to metastasize. ACTs are entirely different lesions from chondrosarcomas grades II and III, which are fully malignant tumors with metastatic potential and poor prognosis [1]. Therefore, treatment strategies are also different. While wide resection is the standard of care for chondrosarcoma grades II-III (regardless of the location) and axial chondrosarcoma grade I, the options for managing ACTs in long bones are currently under debate and no consensus has been reached yet [2–5]. Particularly, ACTs can be treated with curettage with or without local adjuvant therapy for sufficient local control [5]. Cryotherapy, electrocauterization and instillation of chemicals such as phenol and ethanol are the main adjuvant treatment options, with no proven superiority regarding the use of one over the others [6]. Curettage with or without adjuvants shows similar oncological outcomes, better functional scores and lower rates of complications compared to wide resection [6–8]. Alternatively, some reference centers now recommend active surveillance with close radiological follow-up for asymptomatic ACTs, aiming at further reducing the complication rates and morbidity associated with surgery [4, 5].

Fractures and infections were the most common non-oncological complications after curettage reported in previous studies, systematic reviews and meta-analyses [6–11]. However, their occurrence was often established based on retrospectively collected clinical data, and post-operative imaging was not evaluated consistently. In this study, we aimed to assess imaging findings and complications of ACTs of long bones treated with curettage, adjuvants, and either cementation or bone grafting. Our ultimate goals were to familiarize physicians with the post-operative imaging appearance and highlight normal and abnormal findings, complications and their prevalence, which should be considered when opting for a conservative or surgical approach.

## Materials and methods

### Design and population

Our Institutional Review Board approved this retrospective cohort study (study protocol: “RETRORAD”) and waived the need for informed consent. The strengthening the reporting of observational studies in epidemiology (STROBE) checklist was followed [12]. Consecutive patients with ACTs of long bones surgically treated between 2009 and 2022 at our tertiary bone tumor center (IRCCS Orthopedic Institute Galeazzi, Milan, Italy) were considered for inclusion. Information was retrieved through medical records from the oncological orthopedic surgery, pathology and radiology departments. Inclusion criteria were: (i) central ACTs of long bones (defined based on the 2020 edition of the WHO classification [1]) treated with curettage, adjuvant therapy and either cementation or bone grafting, with or without internal fixation; (ii) definitive diagnosis of ACT based on post-surgical pathology; (iii) minimum follow-up of 2 years, including both clinical assessment and imaging evaluation (imaging studies performed as per our follow-up protocol detailed in the second to next paragraph, with all patients having radiographs, CT and MRI scans available for review). Exclusion criteria were locally recurrent lesions and the presence of pathological fractures.

### Surgical procedure

All surgical procedures were performed by expert orthopedic surgeons with 6 to 30 years of experience in bone tumors. In each patient, the tumor was accessed through a cortical window and extensive curettage was carried out. After electrocauterization, phenolization and ethanol irrigation, the cavity was filled with either bone graft (autograft or allograft, based on the size of the cavity) or cement (polymethylmethacrylate), and the cortical window was refashioned to the bone. Prophylactic internal fixation with screws and plates was used to prevent fracturing in most patients [13], also taking into account the location and size of the lesion. All patients were instructed regarding mobilization and weight-bearing, which were supervised by physiotherapists.

### Follow-up

Both radiographs and CT were performed on the same day of surgery for initial reference. Follow-up consisted of physical examination and radiographs obtained at 6 weeks, at 3, 6, 12, 18 and 24 months and then on an annual basis after the procedure. Cross-sectional imaging studies such as MRI or CT were obtained at 6, 12, 18 and 24-month follow-up and then annually. CT was favored over MRI during the first year to detect any post-surgical complication. Subsequently, a choice between these two modalities was based on the presence and extent of internal fixation hardware, which would result in magnetic susceptibility artifacts on MRI.

### Normal imaging findings and complications

All imaging studies (radiographs, CT and MRI) performed between the immediate post-operative period and the last follow-up were independently assessed by a musculoskeletal radiologist with 5 years of experience in bone tumors (S.G.), a general radiologist (A.S.) and a last-year radiology resident (M.S.).

**Fig. 1:**
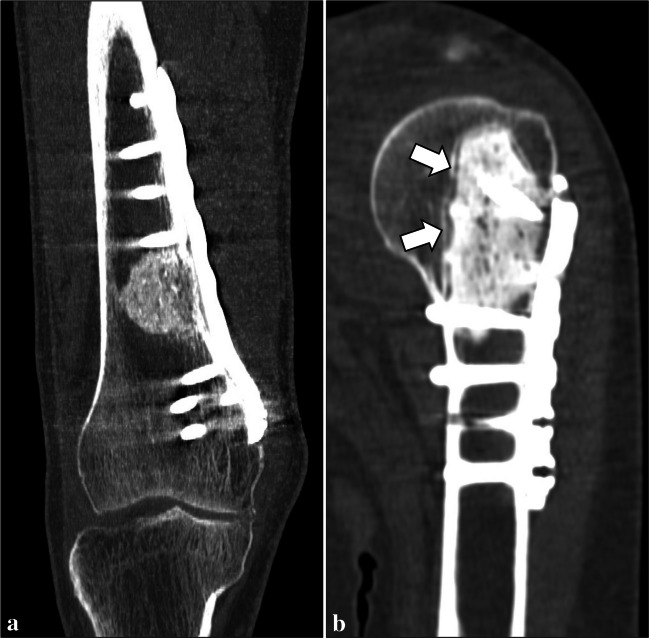
Normal imaging findings after curettage and bone grafting or cementation in a 73-year-old man (**a**) and in a 40-year-old woman (**b**), respectively. After curettage and bone grafting of an ACT of the distal femur, a coronal CT image shows normal graft integration into the host bone (**a**). After curettage and cementation of an ACT of the proximal humerus, a thin uniform radiolucent zone with sclerotic rim (arrows) around the cemented area is normal (**b**)

In ACTs treated with curettage and bone grafting (autograft or allograft), a heterogenous appearance of the graft including areas of lucency, increased density and bridging trabeculation was regarded as normal, if progressive integration into the host bone could be observed (Fig. 1a) [14]. In ACTs treated with curettage and cementation, a thin uniform radiolucent zone (up to 2 mm thick) with sclerotic rim around the cemented area was attributed to cement-related thermal osteonecrosis and regarded as normal if it did not change over time (Fig. 1b) [14].

The following abnormal findings and complications were assessed and recorded by the three independent readers, using a standardized scoring sheet:

- Perioperative bone fracture (occurring because of curettage and/or internal fixation), which was defined as an iatrogenic cortical breakthrough unrelated to the cortical window and detected on the same day of surgery.
- Post-operative bone fracture, which occurred following patient’s discharge.
- Rupture of the fixation hardware, including plates and/or screws.
- Incomplete or absent graft integration into the host bone, which was defined as partial or complete failure of graft integration associated with resorption of the adjacent host bone (cortical and cancellous), in patients treated with curettage and bone grafting.
- Cement loosening, which was defined as bone resorption around the cemented area that exceeded 2 mm and involved both cortical and cancellous bones, in patients treated with curettage and cementation.
- Hardware loosening, which was defined as a lucency exceeding 2 mm around fixation hardware.
- Residual disease or local recurrence.

The most experienced reader also assessed the largest tumor diameter on preoperative images, such as MRI and/or CT. Additionally, information regarding the occurrence of wound infections or need for re-intervention for any reason was retrieved through the medical records.

### Statistical analysis

Statistical analysis was performed using IBM SPSS Statistics 29.0 (IBM Corp., Armonk, NY, USA). In addition to the post-surgical complications assessed on imaging studies or retrieved through the medical records, the following data were collected and included in the analysis: age, gender, tumor location, filler of the curetted cavity and use of internal fixation hardware. Continuous variables were reported as medians and 1^st^-3^rd^ interquartile ranges (IQR), and categorical variables were reported as absolute values and percentages. Differences between patients with and without post-surgical complications were evaluated using Chi-square and Fisher’s exact tests for categorical variables, and non-parametric Mann–Whitney U-test for continuous variables. A two-sided p-value < 0.05 indicated statistical significance. Inter-reader agreement was assessed using Fleiss’ kappa. Kappa statistics was interpreted as follows: K ≤ 0 indicated no agreement, 0 < K ≤ 0.20 indicated slight agreement, 0.20 < K ≤ 0.40 indicated fair agreement, 0.40 < K ≤ 0.60 indicated moderate agreement, 0.60 < K ≤ 0.80 indicated substantial agreement, and 0.80 < K ≤ 1 indicated almost perfect agreement, respectively [15].

## Results

### Characteristics of the study cohort

Out of 109 patients with central ACTs of long bones who were surgically treated between 2009 and 2022, 41 were excluded. The reasons for exclusion were surgical treatment consisting of wide resection rather than curettage (n = 9), follow-up shorter than 2 years or unavailability of post-operative imaging (n = 29), locally recurrent lesion (n = 1) and pathological fractures (n = 2). Sixty-eight patients were finally included in this study (23 men and 45 women, with median [IQR] age of 53 [45–60] years). ACTs were located in the femur (n = 35), fibula (n = 1), humerus (n = 28) and tibia (n = 4). The median (IQR) largest tumor diameter was 35 (28–55) mm. A flowchart of the patients’ selection process is shown in Supplementary Fig. 1.

All lesions were treated with curettage and adjuvant therapy consisting of electrocauterization, phenolization and ethanol irrigation. After curettage and instillation of adjuvants, cementation was performed between 2009 and 2017, and bone grafting was performed between 2012 and 2022, respectively. Particularly, bone graft and cement were used to fill the curetted cavity in 53 (77.9%, including autologous [n = 6] and homologous [n = 47] grafts) and 15 (22.1%) patients, respectively. Prophylactic internal fixation was performed in 63 (92.7%) patients. The median (IQR) follow-up duration was 42 (30–64) months.

### Imaging findings and post-surgical complications

**Fig. 2:**
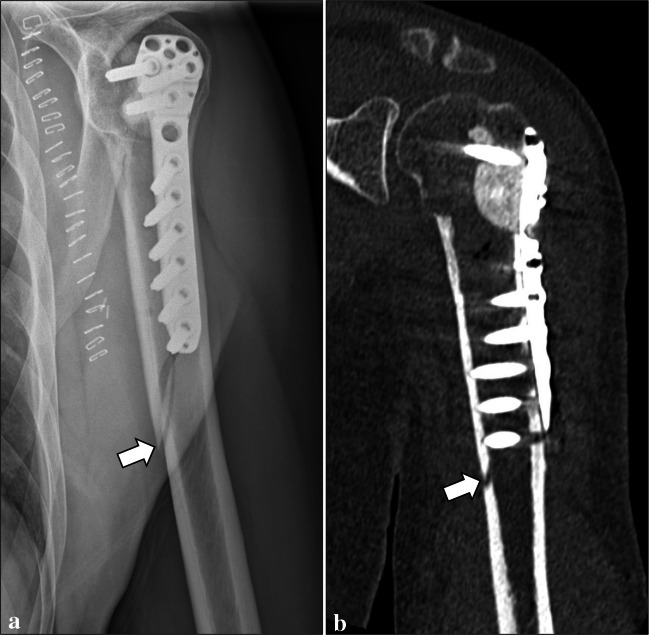
Perioperative bone fracture in a 65-year-old man. A non-displaced fracture of the humeral diaphysis (arrows) was noted after curettage of an ACT of the proximal humerus, bone grafting and internal fixation on radiograph (**a**) and CT (**b**)

**Fig. 3:**
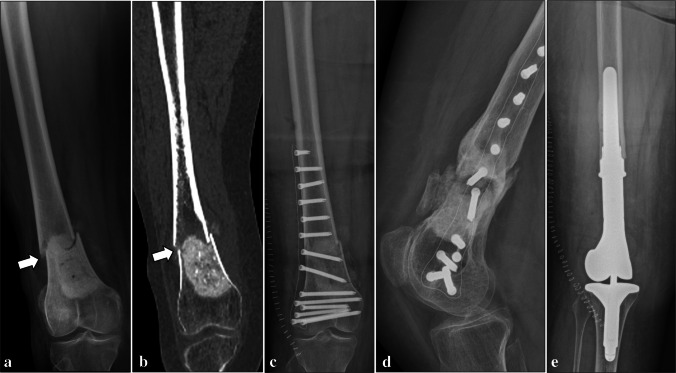
Post-operative bone fracture in a 53-year-old woman. Bone fracture (arrows) occurred 2 months after curettage of an ACT of the distal femur, as shown on radiograph (**a**) and CT (**b**). Treatment consisted of internal fixation using screws and plate (**c**). However, non-union occurred (**d**), and wide resection and reconstruction with knee megaprosthesis were finally needed (**e**)

**Fig. 4:**
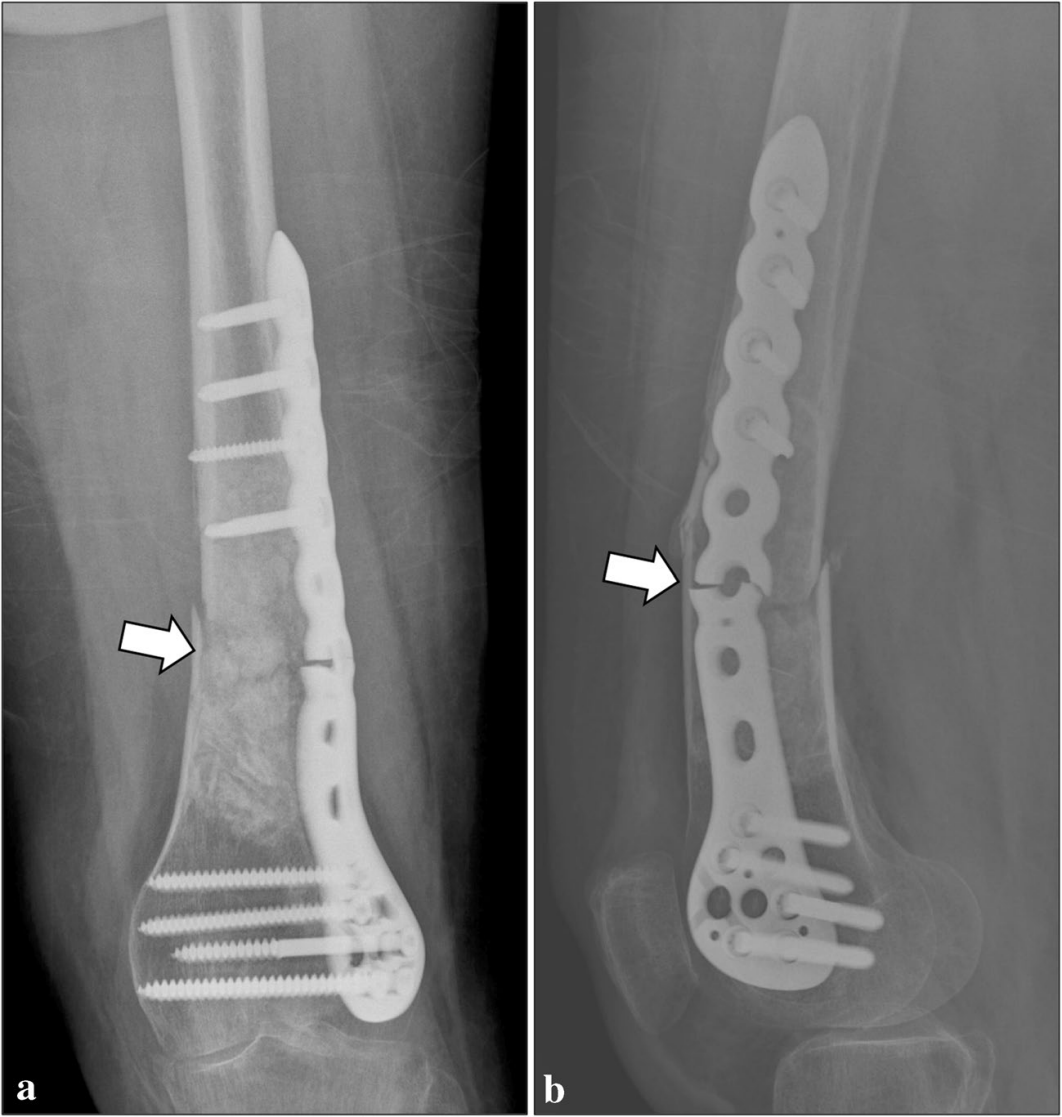
Post-operative bone fracture and plate rupture in a 55-year-old woman. Bone fracture and plate rupture (arrows) occurred 17 months after curettage of an ACT of the distal femur, bone grafting and internal fixation, as shown on frontal view (**a**) and lateral view (**b**) radiographs. Fixation surgery was performed using a longer plate and progressive healing occurred thereafter (not shown)

Normal imaging findings were identified at follow-up examinations in 45 patients (66.2%). Table 1 summarizes the post-operative complications detected on imaging in our series. Perioperative bone fractures (Fig. 2) were identified in 2 patients (2.9%) on the same day of surgery. Both had a non-displaced fracture of the humeral diaphysis, which healed after conservative treatment. Post-operative bone fractures (Figs. 3 and 4) were found in 6 (8.8%) patients, which occurred between 6 days and 17 months after patient’s discharge. Among them, two diaphyseal fractures of the distal femur were surgically treated. In detail, one of them was treated with internal fixation using screws and plate (no internal fixation was performed at the time of curettage), was complicated by non-union, and finally required revision surgery consisting of wide resection and reconstruction with knee megaprosthesis (Fig. 3). The other femoral diaphyseal fracture was treated with revision of the fixation hardware (internal fixation was performed at the time of curettage) using a longer plate and showed progressive healing after revision surgery. The remaining post-operative fractures included an avulsion fracture of the greater trochanter of the femur, two avulsion fractures of the greater tuberosity and a diaphyseal fracture of the humerus, which showed no to minimal displacement and were treated conservatively. Rupture of the fixation hardware (Fig. 4) was identified in 1 patient (1.6%, out of those treated with internal fixation), where plate rupture was observed in association with a fracture of the femoral diaphysis. No rupture of the screws was reported in our series.

**Table 1:** Complications detected on imaging in ACTs of long bones treated with curettage, adjuvants, and either bone grafting or cementation. Lesion size (maximum diameter) is expressed as a single value if the complication occurred only in one patient, or median (IQR) if it occurred in more than one patient

Complications	Prevalence	Lesion size	Location	Cavity filler	Internal fixation	Complication management
Perioperative fracture	2.9% (2/68)	53 (50–55) mm	Humeral diaphysis (*n* = 2)	Bone graft (*n* = 1) Cement (*n* = 1)	Yes (*n* = 2)	Conservative
Post-operative fracture	8.8% (6/68)	37 (28–48) mm	Femoral diaphysis (*n* = 2) Femoral greater trochanter (n = 1) Humeral diaphysis (*n* = 1) Humeral greater tuberosity (*n* = 2)	Bone graft (*n* = 6)	Yes (*n* = 5) No (n = 1)	Conservative (n = 4, humerus and greater trochanter) Surgery (n = 2, femoral diaphysis)
Hardware rupture	1.6% (1/63*)	58 mm	Femur	Bone graft	Yes	Surgery
Incomplete bone graft integration	24.5% (13/53**)	31 (28–55) mm	Femur (*n* = 4) Humerus (*n* = 8) Tibia (*n* = 1)	Bone graft (*n* = 13)	Yes (*n* = 13)	Conservative
Cement loosening	26.7% (4/15***)	38 (31–46) mm	Fibula (*n* = 1) Humerus (*n* = 3)	Cement (*n* = 4)	Yes (*n* = 3) No (*n *= 1)	Conservative
Hardware loosening	6.3% (4/63*)	54 (44–71) mm	Femur (*n* = 1) Humerus (*n* = 3)	Bone graft (*n* = 3) Cement (*n* = 1)	Yes (*n* = 4)	Conservative
Residual tumor	1.5% (1/68)	40 mm	Humerus	Cement	No	Follow-up

* Treated with internal fixation

** Treated with bone grafting

*** Treated with cementation

**Fig. 5:**
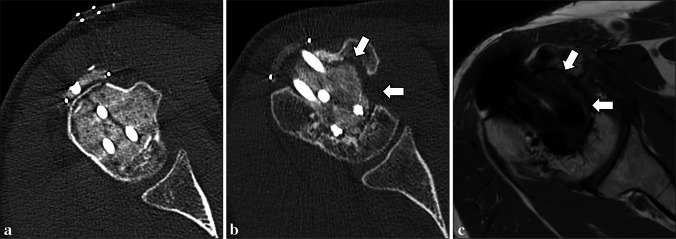
Incomplete bone graft integration in a 54-year-old man. After curettage of an ACT of the proximal humerus, the cavity was entirely filled with bone graft on the CT scan performed on the day of surgery (**a**). Failure of graft integration occurred along with resorption of the adjacent host bone (arrows), as shown on CT (**b**) and T1-weighted MRI (**c**) images at 2-year follow-up

**Fig. 6:**
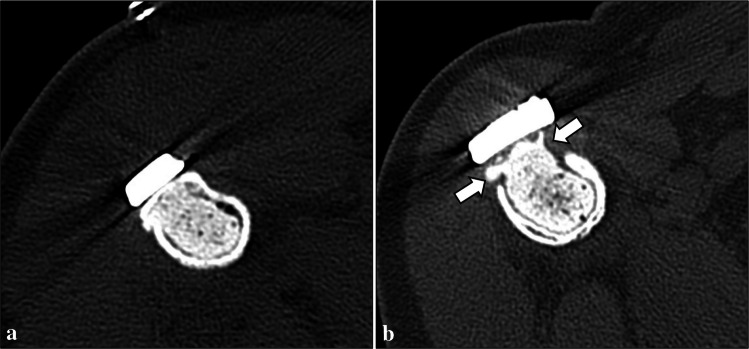
Cement loosening in a 51-year-old woman. After curettage of an ACT of the proximal humerus, the cavity was entirely filled with cement on the CT scan performed on the day of surgery (**a**). Cortical and cancellous bone resorption (arrows) progressively occurred around the cemented area after surgery, as shown on CT at 2-year follow-up (**b**)

**Fig. 7:**
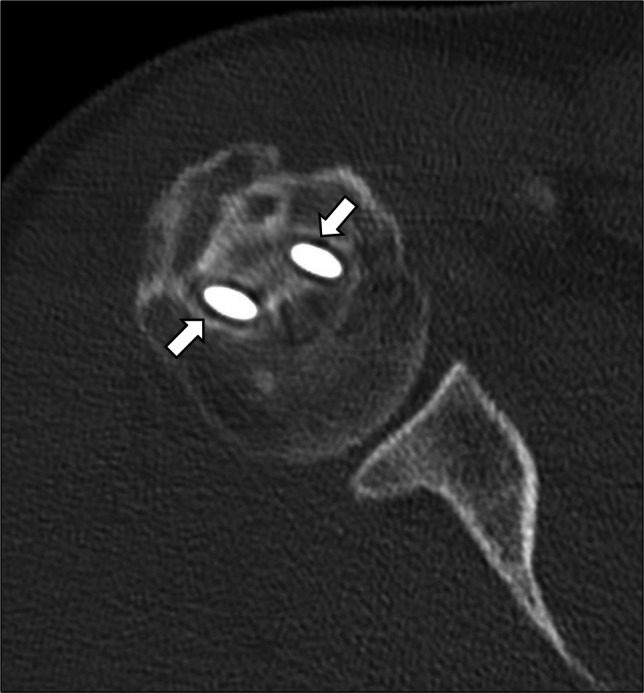
Hardware loosening in a 42-year-old man. After curettage of an ACT of the proximal humerus, bone grafting and internal fixation, CT showed a lucency exceeding 2 mm (arrows) around the screws at 2-year follow-up

Incomplete bone graft integration (Fig. 5) was described in 13 patients (24.5%, out of those treated with bone grafting). In all cases, resorption of the adjacent host bone was associated to the graft failure, which was deemed as partial because incorporation into the host bone was present in one third-to-half of the graft surface. Completely absent bone graft integration was not reported in any case from our series. Cement loosening (Fig. 6) was reported in 4 patients (26.7%, out of those treated with cementation), where both cancellous and cortical bone resorption was identified around the cemented area. In all cases of failure of bone graft integration or cement loosening, residual/recurrent tumor was ruled out based on both CT and MRI. Hardware loosening (Fig. 7) was found in 4 patients (6.3%, out of those treated with internal fixation), involving one or more screws.

**Fig. 8:**
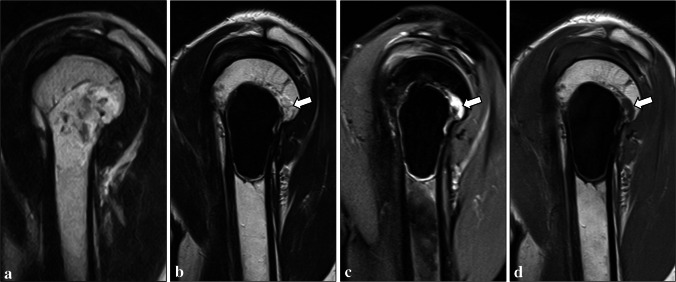
Residual tumor in a 57-year-old woman. On preoperative sagittal T2-weighted MRI sequence, an ACT of the proximal humerus could be seen (**a**). After curettage and cementation, a residual cartilaginous lesion (arrows) was identified on sagittal T2-weighted MRI sequences without (**b**) and with (**c**) fat suppression and on sagittal T1-weighted sequence (**d**), respectively. It was monitored through repeated MRI scans and showed no increase in size during a follow-up of 5 years

Residual disease (Fig. 8) was reported in 1 patient (1.5%) with an ACT of the proximal humerus. In this case, a residual cartilaginous lesion was identified post-operatively and then monitored through repeated MRI scans, with no increase in size detected during a follow-up of 5 years. No tumor recurrence was observed in our series.

In our medical records, no case of post-surgical infection was reported. In addition to the two cases of surgically treated post-operative fractures, a re-intervention was performed in 13 patients (20.6%, out of those treated with internal fixation) to remove the fixation hardware because of complaints related to it, such as pain and movement limitation. In none of the patients with incomplete bone graft integration or cement loosening, the fixation hardware was removed.

Among the post-surgical complications, incomplete bone graft integration and cement loosening were associated with tumor location in the upper extremity (p = 0.023). Tumor location was not associated with any of the remaining imaging findings and complications (p ≥ 0.166). Age (p ≥ 0.210), gender (p ≥ 0.546), tumor size (p ≥ 0.146), filler of the curetted cavity (p ≥ 0.221) and the use of internal fixation (p ≥ 0.074) were not associated with any of the evaluated imaging findings and complications. Inter-reader reliability ranged between moderate and excellent for all findings assessed by the three radiologists, as detailed in Table 2.

**Table 2:** Inter-reader agreement for all evaluated imaging features

Complications detected on imaging studies	Fleiss’s K	95% confidence interval
Perioperative bone fracture	0.852	0.715–0.989
Post-operative bone fracture	0.671	0.534–0.809
Hardware rupture	1.000	0.863–1.000
Incomplete bone graft integration or cement loosening	0.622	0.485–0.759
Hardware loosening	0.522	0.379–0.665
Residual/recurrence	1.000	0.863–1.000

## Discussion

In the present study, we assessed post-operative findings and complications on imaging of ACTs of long bones treated with curettage, adjuvant therapy, and either cementation or bone grafting. In our series of 68 patients with available clinical and radiological follow-up of at least 2 years, peri- and post-operative bone fractures (overall 11.7%), incomplete bone graft integration (24.5%) and cement loosening (26.7%) were the most frequent abnormalities. The rate of bone fractures was in line with previous studies [10, 16]. Previous studies did not focus on failure of bone graft integration or cement loosening, as most follow-up information was obtained through medical records rather than a re-assessment of imaging examinations, as done in our study. In our series, incomplete bone graft integration and cement loosening were associated with tumor location in the humerus (p = 0.023). The clinical significance of incomplete bone graft integration and cement loosening is matter of debate. Indeed, one may argue that host bone resorption around the curetted cavity (filled with either bone graft or cement) could result in an increased fracture risk. However, follow-up duration should be longer than this study to ascertain whether this association exists. In our series, none of the patients with graft integration failure or cement loosening underwent hardware removal surgery to prevent the risk of fracturing.

Among the least common complications reported in our study, fixation hardware rupture (1.6%) occurred in association with bone fracturing, and fixation hardware loosening (6.3%) occurred in association with bone graft failure or cement loosening, respectively. Our rate of residual disease (1.5%) was lower compared to previous studies reporting up to 14% residual rate [10]. Finally, we had no case of post-surgical infection. In our series, a re-intervention was needed in 2 patients with post-operative bone fracture (2.9%) and 13 patients with local pain and movement limitation (20.6%), where fracture fixation and hardware removal were performed, respectively. Our re-intervention rate was in line with previous studies [9, 10].

Overall, our rate of post-operative complications was not negligible, as they occurred in one third of patients. Thus, the radiologists should be vigilant to detect the findings observed in our cohort. In addition, based on our findings and recent insights on the natural course of ACTs with low growth rates and no reported transformation into chondrosarcoma grade II or higher [17–22], future research may refine the treatment approach for ACTs, balancing the overall benefits and complications of curettage against surveillance strategies.

Some limitations of this study should be addressed. First, our study design was retrospective, which is however common in skeletal oncology, as bone tumors are low prevalent and retrospective design allowed us to include a relatively large cohort of patients with follow-up data available. Second, this study included patients treated between 2009 and 2022, and the indications for curettage changed during this period. Indeed, since WHO reclassified chondrosarcoma grade I of the appendicular skeleton as “ACT” in 2013 [23] (further refining the terminology in 2020 [1]), a progressive shift from surgery to active surveillance has been seen, particularly for patients without symptoms [4, 5]. At our institution, after 2013, the choice between curettage and active surveillance depended on several factors, including patients’ symptoms, concerns and compliance to follow-up visits. Third, in this study, all patients were treated with curettage followed by electrocauterization and instillation of phenol and ethanol, as per clinical practice at our institution. Thus, no comparison was performed with other adjuvant options, such as cryotherapy. Fourth, functional results according to the Musculoskeletal Tumor Society scores were not included, as they were not adequately collected on a prospective basis. Finally, this study focused on short-to-mid-term follow-up and long-term complications such as secondary osteoarthritis [24] were not evaluated.

In conclusion, radiologists interpreting follow-up radiographs and cross-sectional imaging studies must be familiar with the normal appearance of curetted ACTs and recognize post-operative abnormal findings and complications, which were found to occur in one third of patients from our cohort.

## Supplementary Information

Below is the link to the electronic supplementary material.

Fig. 9 (PNG 84.8 KB)

ESM 1 (TIF 16.3 MB)

## Data Availability

Deidentified datasets will be published in the online repository Zenodo (Zenodo.org) upon manuscript publication.
