# Supplementary figures and images for: Imaging findings and complications after curettage of atypical cartilaginous tumors in long bones: a retrospective single-center cohort study
Source: Skeletal Radiol. 2025 Oct 29;55(3):641–50. doi: 10.1007/s00256-025-05061-7 (PMC12847113; doi:10.1007/s00256-025-05061-7)

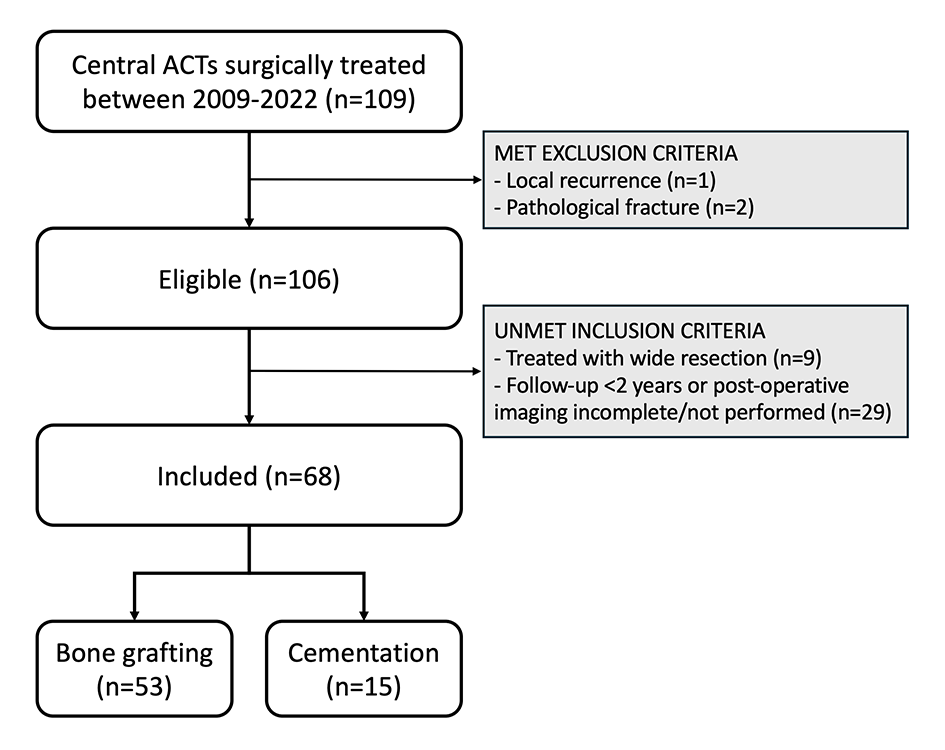

Supplement: Supplementary file 1 — (PNG 84.8 KB) [file 256_2025_5061_Fig9_ESM.png]

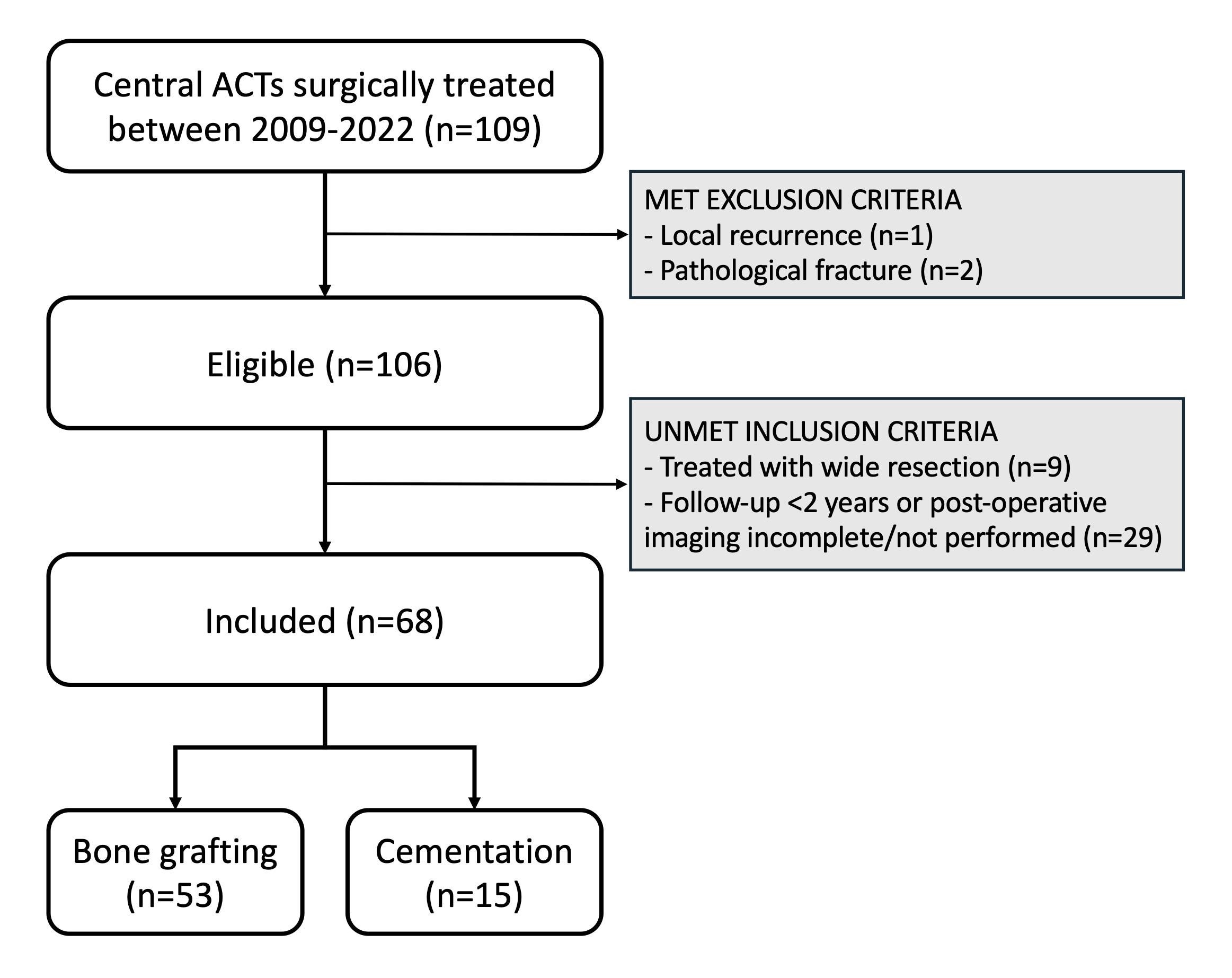

Supplement: Supplementary file 2 — (TIF 16.3 MB) [file 256_2025_5061_MOESM1_ESM.tiff]
